# Acute Abdomen Revealing an Unusual Case of Intra-Abdominal Testicular Torsion

**DOI:** 10.1155/2019/5815036

**Published:** 2019-12-28

**Authors:** Maazou Halidou, Amadou Roua, Ibrahim Amadou Magagi, Harissou Adamou, Ousseini Adakal, Mohamed Rouga, Soumana Amadou, Rachid Sani

**Affiliations:** ^1^Department of Urology, Zinder National Hospital, Zinder, Niger; ^2^Faculty of Health Sciences, University of Zinder, Zinder, Niger; ^3^Department of Urology, Regional Hospital Center of Maradi, Maradi, Niger; ^4^Department of General Surgery, Zinder National Hospital, Zinder, Niger; ^5^Department of General Surgery, Regional Hospital Center of Maradi, Maradi, Niger; ^6^Faculty of Health Sciences, Dan Dicko Dankoulodo University, Maradi, Niger; ^7^Department of Surgery and Surgical Specialties, Faculty of Health Sciences, University of Niamey, Niamey, Niger

## Abstract

*Introduction*. Intra-abdominal testicular torsion is a rare event. We report hereby our experience of the management of a spermatic cord twist on intra-abdominal testis discovered during an acute surgical abdomen. *Case Presentation*. This was a 42-year-old patient admitted to the emergency department for abdominal pain that had been evolving for a week. The physical examination showed tenderness and guarding in the left iliac fossa with an empty ipsilateral hemiscrotum. Complementary examinations led to the discovery of an intra-abdominal left-lateral mass. The laparotomy found a whitish mass with areas of infarction, which was resected. Anatomopathological examination of the operative specimen identified it as a testis with atrophy of germ cells and necrotic areas without evidence of malignancy. *Conclusion*. Intra-abdominal testicular torsion should be considered in case of patients with an acute surgical abdomen with vacuity of one of the bursae.

## 1. Introduction

The cryptorchidism is defined as an undescended testis into the scrotum [1–5]. It affects 1–5% of term births and between 1.1 and 45% of premature infants [1, 6, 7]. The main complications of this condition are testicular torsion, malignancy, infertility, and exposure to trauma [2, 6, 7]. The intra-abdominal position of the testis is rare and must be evoked ahead of any vacuity of the scrotum with a nonpalpable testis in the inguinal canal [3]. Indeed, in the proportion of impalpable testes (20%), 50–60% are intra-abdominal [1, 3]. Intra-abdominal testicular torsion is a rare surgical emergency involving the functional prognosis of the testis [6, 8]. It is manifested by an atypical clinical picture dominated by an abdominal symptomology which is a source of diagnostic delay [2, 9, 10]. Its management is also controversial when the testis is viable [2]. We report here our experience of the management of spermatic cord torsion on intra-abdominal testis discovered during an acute surgical abdomen.

## 2. Case Report

**Figure 1:**
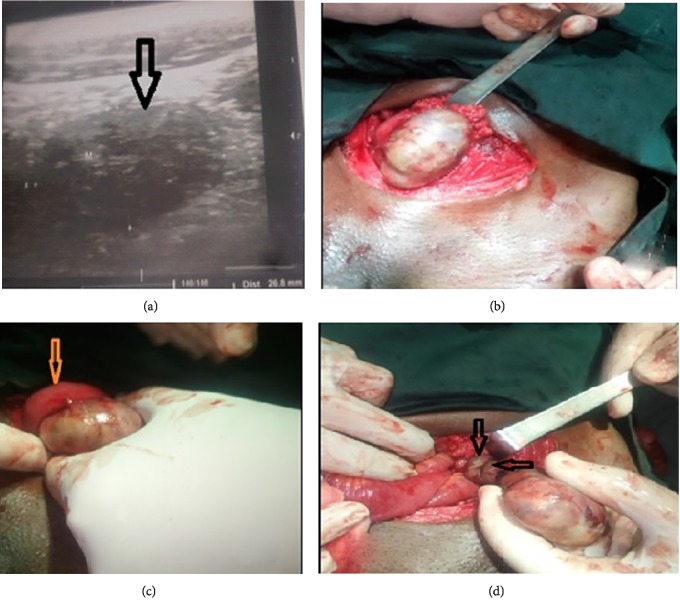
(a) Ultrasound (left lateral vesical mass); (b) mass of externalised testicular appearance; (c) intestinal loop adherent to the mass; (d) pedicle twisting.

A 42-year-old man, married with two children, was admitted to the emergency department for left iliac fossa pain radiating to the flank, which had been evolving for a week without fever. The pain was previously dull, little sensitive to the usual analgesics, with periods of remission. It progressively worsened. After initial care in a peripheral health facility without improvement, the patient was referred to the regional hospital. Clinical examination noted left iliac fossa tenderness with guarding, free hernial orifices, and a vacant left hemiscrotum with a right normal testis; the rectal examination found lateralized pain on the left side of the Douglas pouch. The rest of the somatic examination was unremarkable. The blood count indicated leukocytosis at 12,000 elements/mm^3^ and a normal hemoglobin level. Ultrasound revealed a heterogeneous left-vesical mass of 43 mm × 32 mm in diameter (Figure 1(a)). After a standard preoperative assessment, surgical exploration by laparotomy was decided. Pfannenstiel incision was made and revealed a pedunculated whitish mass with areas of infarction, testicular-like, adhering to an intestinal loop (Figures 1(b) and 1(c)). The pedicle was twisted twice (Figure 1(d)). Resection of the mass was performed. Considering the elements of the clinical examination, the probable diagnosis of torsion of the spermatic cord on intra-abdominal testis was retained. Histopathological examination of the specimen confirmed the testicular nature of the mass and noted germ cells atrophy with necrotic areas and no malignancy sign. The tumor markers: *β*HCG (choriogonadotrophic hormone), *α*FP (alpha fetoprotein), and LDH (Lactodeshydrogenase), measured postoperatively were normal. Postoperative recovery was uneventful. The patient was discharged at postoperative day 5.

## 3. Discussion

Intra-abdominal testicular torsion is a rare event [5, 9, 10]. Few cases have been described in the literature to date, and the true impact of this entity remains poorly understood [9–11]. Gerster was the first author to report a similar case in 1898 [8, 12, 13]. An intra-abdominal testis has a greater risk of torsion than that which is normally descended, because of lack of anatomical attachment structures, the absence of gubernaculum, the size of the spermatic cord which is not in adequacy with the size of the testicle which is often atrophic [2, 10].

It is well established that both cryptorchidism and testicular ectopia increase the risk of malignant degeneration. The risk of cancer is 35–40 times higher compared to the normal subject due to higher abdominal temperature than scrotal one [8, 9]. Indeed it is reported that 50%–64% of patients with testicular tumors have an undescended testicle [10, 14]. Cancerization also increases the risk of torsion due to increased testicular volume by the neoplastic process [2, 5, 14]. In our observation, the tumor markers were normal and the pathological examination did not find any sign of malignancy.

The torsion of the testis in intra-abdominal position may be asymptomatic or may lead to nonspecific signs including irritability, nausea, and vomiting, constipation, atypical abdominal pain, hematuria, or even shock, which can be misdiagnosed as appendicitis or diverticulitis [7, 9, 11, 12]. Sometimes it can manifest as an abdominal mass [8]. Diagnostic suspicion is stronger when the clinical examination finds vacant bursae associated with acute abdominal pain [5]. The ultrasonography remains insensitive in the detection of the testis in the intra-abdominal position [5, 12], even if in our case the ultrasound was contributive; this is probably due to the large volume of the testis. CT scan or magnetic resonance imaging gives better results in localization of the testis in this situation [5, 13], but those examinations are not available in current practice in our context. The search for impalpable testicles is more relevant to a laparoscopic exploration which is the gold standard in this field or a conventional exploratory surgery [8, 12].

The management of testicular torsion on an intra-abdominal testis remains controversial when the testis is viable; some authors advocate orchiectomy because of the high risk of malignant degeneration [9, 10, 15], while other authors advocate orchidopexy in children under 2 years, or in adults who refuse orchiectomy for psychological or aesthetic reasons [9, 10]. This orchidopexy should be associated with clinical monitoring and regular tumor marker assays [9, 10].

In our Nigerien context with limited resources, the discovery of an intra-abdominal testis is often fortuitous, or in an acute surgical abdomen chart and in adulthood, an orchiectomy should be the treatment of choice, as recommended by experts [1].

## 4. Conclusion

Intra-abdominal testicular torsion is rare and often presents as a misleading clinical picture. However, this diagnosis should be considered in a subject with acute abdominal pain with one or two vacant bursae. The orchiectomy remains the recommended treatment because of the significant risk of malignant degeneration that increases with age.
